# Prolonged complete response after treatment withdrawal in HER2-overexpressed, hormone receptor-negative breast cancer with liver metastases: the prospect of disappearance of an incurable disease

**DOI:** 10.1186/1471-2407-14-690

**Published:** 2014-09-22

**Authors:** Erika Viel, Flavie Arbion, Catherine Barbe, Philippe Bougnoux

**Affiliations:** Centre Hospitalier Universitaire Bretonneau, Tours, France; INSERM, U1069, Nutrition Croissance et Cancer, Faculté de Médecine, Université François-Rabelais, Tours, France

**Keywords:** Breast, Cancer, Metastatic, Cure, Complete response

## Abstract

**Background:**

Metastatic breast cancer has consistently been viewed as a non-curable disease. Specific palliative treatments such as chemotherapy and hormone therapy have resulted in a mean overall survival of approximately 30 months. While cases of prolonged complete response have been reported with hormone or trastuzumab monotherapy, rendering metastatic breast cancer a chronic disease, any treatment withdrawal has ineluctably led to relapse. Prolonged remission without any anti-cancer treatment has never been reported to our knowledge.

**Case presentation:**

We report here the unique observation of the spontaneous evolution of two breast cancer patients with synchronous liver metastases who decided to stop trastuzumab after achieving complete response. They were Caucasian women with synchronous liver metastatic breast carcinoma. Both breast cancers reached skin and regional lymph nodes. There were several liver metastases in both patients. They received surgery, radiotherapy and chemotherapy combined with trastuzumab. They decided to stop their treatment, despite guidelines. After a follow-up longer than 20 months, they did not relapse clinically, radiologically, and biologically.

**Conclusion:**

This findings question the belief of the unavoidability of recurrence of metastatic breast cancer, specifically in the liver. It opens up the unprecedented possibility of a cure-like state in exceptional and probably special cases.

## Background

Metastatic breast cancer has consistently been considered as a non curable disease, which needs specific palliative treatments in order to minimize symptoms and allow patients to live with their disease [1]. With systemic treatments such as chemotherapy and hormonal therapy in patients whose tumor expresses steroid hormone receptors, mean overall survival has been ranging between 27.2 and 32.3 months [2, 3]. The addition of anti-HER2 (Human Epidermal Growth factor 2) molecules such as trastuzumab in patients with overexpression and/or gene amplification of HER2 in tumors, has deeply modified the evolution of the metastatic disease, leading to an increase in OS (Overall Survival) to 37.6 month [4]. Even in visceral metastases, such as liver metastases, where OS has been particularly short when patients are not amenable to surgery (19 to 26 months) [5] trastuzumab use has led to a dramatic improvement, up to 32 months [6].

Prolonged complete responses have been reported in steroid receptor positive metastatic breast cancer under endocrine treatments. However, any interruption of this endocrine treatment ineluctably leads to relapses. Similarly, in steroid receptor negative, HER2 positive metastatic breast cancer patients receiving anti-HER2 monotherapy, there have been several observations of sustained complete remission and long-term survival [7, 8], and for the same reasons, it is thought that any interruption should lead to relapses. In addition, guidelines presently recommend that anti-HER2 treatments should be provided indefinitely, i.e.: never interrupted as long as there is no toxicity or tumor progression [9]. However, the main difference between the two situations is that trastuzumab may have a cytotoxic action, contrary to endocrine treatments [10, 11]. This potential cytotoxicity raises the question of the palliative or curative nature of trastuzumab treatment, and to our knowledge, a persistent complete response after total discontinuation of trastuzumab has never been reported in this setting.

We report here the cases of two patients with negative steroid receptors, HER2-overexpressed breast carcinoma, who presented with synchroneous liver metastases. They received loco-regional and systemic treatments, and achieved complete response. Contrary to recommendations, they eventually decided to stop anti-HER2 treatment, and did not relapse. We report the unique observation of their spontaneous evolution.

## Case presentation

**Figure 1:**
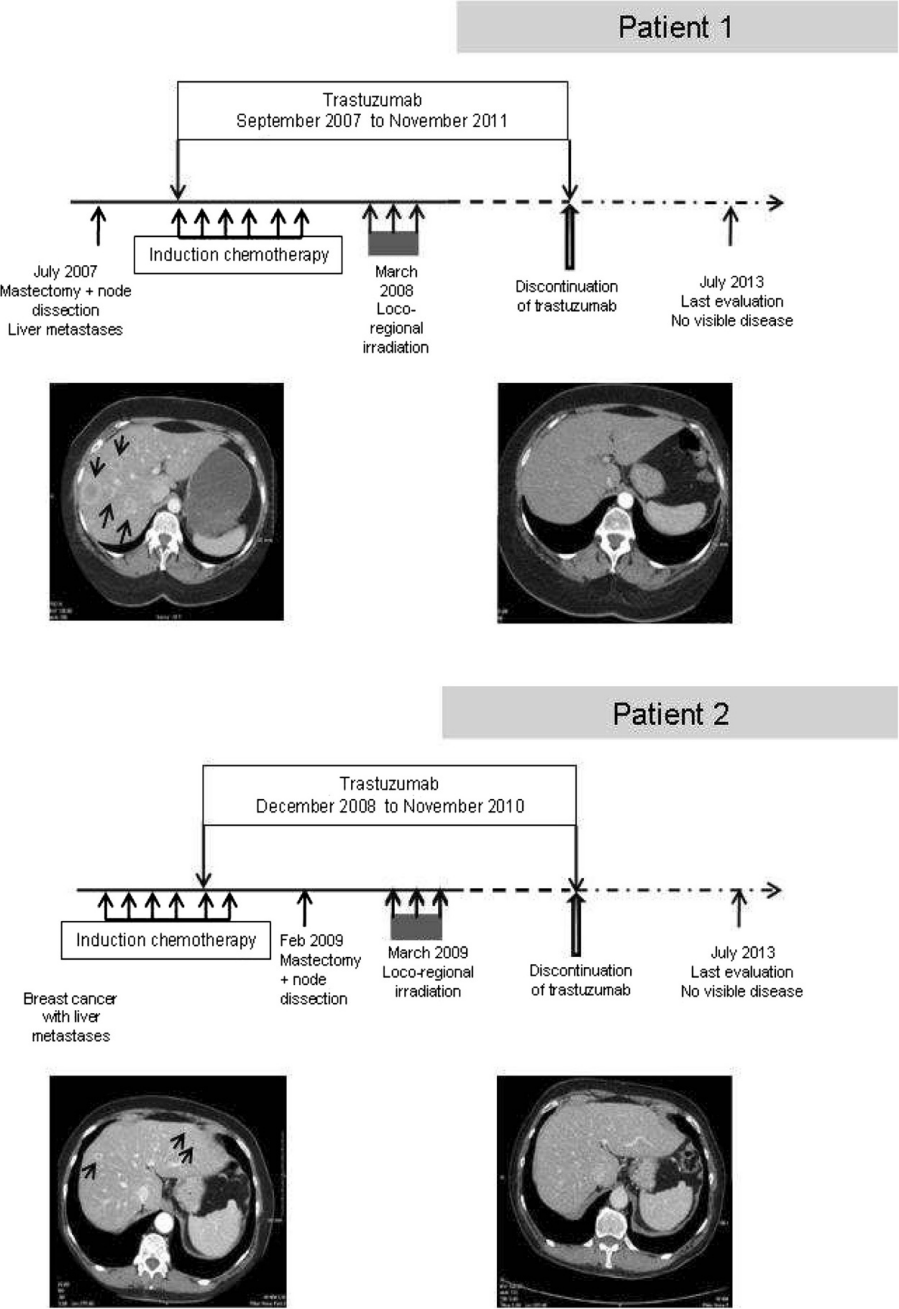
**Clinical features and evolution of patients.** Loco-regional and systemic anti-cancer treatments of patients. Pathognomonic CT-scan pictures of liver metastases at presentation (left), and complete response (right).

**Figure 2:**
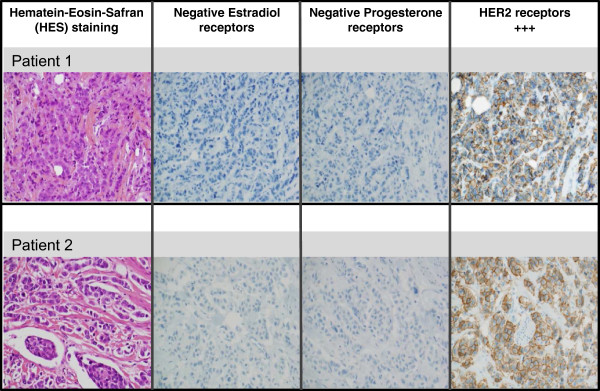
**Pathological and immunohistochemical characterisation of tumors.** Pathologic analyses were performed on paraffin-embedded tissue blocks. Haematoxylin and eosin staining (HES) confirmed invasive SBR3 carcinomas. No staining was detected after estrogen receptor (1D5, DAKO) and progesterone receptor (PgR 636 DAKO) branding compared to positive and negative controls. Using the A485 polyclonal antibody (DAKO), a strong brown complete membrane staining was detected in more than 30% tumor cells in both patients.

A 54-year-old woman and a 70-year-old woman were referred to the Tours University Hospital Cancer Ward with locally advanced breast cancer, a skin-connected large breast cancer in one and an inflammatory breast cancer in the second one, in 2007 and 2008, respectively. Both had positive axillary lymph nodes. Several pathognomonic images of synchronous liver metastases were present on their CT-scans [12] (Figure 1). Pathology analysis of the breast tumors (biopsy or surgery) confirmed invasive ductal carcinomas, with a SBR grade of 3, negative for steroid receptors but HER2-positive on immunohistochemistry (Figure 2). Both patients had a mastectomy and a minimal axillary lymph node resection as several reports suggest that locoregional treatment of the primary tumor improves outcome in women with stage IV breast cancer at diagnosis [13, 14]. The first patient had a hemorrhagic tumor, and the second patient required a rapid complete locoregional treatment subsequent to induction chemotherapy. Both patients had radiation therapy of the chest wall. This included the supra clavicular area for the second patient (Figure 1).

The first patient received 8 cycles of docetaxel (100 mg/m^2^) combined with trastuzumab (6 mg/kg every 3 weeks). Trastuzumab was continued as maintenance therapy. Complete response was obtained in the liver 23 months after diagnosis. She decided to cease taking trastuzumab after 50 months of treatment. At 30 months’ follow-up after withdrawal of trastuzumab, she remains in complete response biologically and radiologically (Figure 1).

The second patient received 4 cycles of epirubicin-containing chemotherapy (FEC 100 regimen), then 2 cycles of docetaxel (100 mg/m^2^) combined with trastuzumab (6 mg/kg every 3 weeks). Complete response was documented by pathology results in the resected breast. Radiology results showed more than 50% partial response of liver metastases at the time of breast surgery. Trastuzumab maintenance followed this induction treatment. Complete liver response was achieved after 7 months’ trastuzumab maintenance, i.e.: 1 year after diagnosis. The patient decided to stop trastuzumab treatment after 24 months of maintenance. She has not received any specific anti-cancer treatment for 42 months, and still displays a complete biological and radiological response (Figure 1).

In HER2-overexpressed metastatic breast cancer, median OS has been reported to be close to 25 months, and median time to progression (TTP) approximately 12.4 months [7], despite the use of specific treatments. In our two cases, although all treatment had been withdrawn for 30 and 42 months, no relapse occurred. This appears quite remarkable and raises the question of the already suggested [15] possible eradication of these presentations of metastatic breast cancer as a consequence of the medical treatment.

It is not known whether these results might be specific to this type of cancer, i.e.: HER2-overexpressed, steroid receptor-negative breast carcinoma, as already evoked [15], since this has never been reported with other pathological features. This case-report is not intended to establish whether a cause-and-effect relationship exists between those excellent clinical results and trastuzumab administration. One should wonder if the theoretical cytotoxicity of trastuzumab might potentially lead to disappearance or dormancy of any cancer clone, the commonly accepted cause of relapse [16].

## Conclusions

Other similar cases may not have been identified to date because of current guidelines [8]. This short paper may generate other reports which may allow the constitution of a cohort of similar cases. Predicting among patients in complete response which patients are most likely to do well after trastuzumab withdrawal remains a challenge. Finally, the present situation raises questions regarding the belief in the unavoidability of recurrence of metastatic breast cancer, specifically in the liver. It opens up the unprecedented possibility of a cure-like state in exceptional cases.

### Consents

Written informed consent was obtained from each patient for publication of this Case report and any accompanying images. A copy of the written consents is available for review by the Editor of this journal.

## Abbreviations

OS: Overall survival
HER2: Human epidermal growth factor receptor 2
TTP: Time to progression.
